# Cerebrospinal fluid levels of proenkephalin and prodynorphin are differentially altered in Huntington’s and Parkinson’s disease

**DOI:** 10.1007/s00415-022-11187-8

**Published:** 2022-06-23

**Authors:** Peggy Barschke, Samir Abu-Rumeileh, M. H. D. Rami Al Shweiki, Lorenzo Barba, Federico Paolini Paoletti, Patrick Oeckl, Petra Steinacker, Steffen Halbgebauer, Lorenzo Gaetani, Jan Lewerenz, Albert Christian Ludolph, Georg Bernhard Landwehrmeyer, Lucilla Parnetti, Markus Otto

**Affiliations:** 1 Department of Neurology, Ulm University Hospital, 89081 Ulm, Germany; 2 Department of Neurology, Martin-Luther-University Halle-Wittenberg, 06120 Halle (Saale), Germany; 3 Section of Neurology, Department of Medicine and Surgery, University of Perugia, 06132 Perugia, Italy; 4 German Center for Neurodegenerative Diseases (DZNE e.V.), 89081 Ulm, Germany

**Keywords:** Biomarkers, Cerebrospinal fluid, Huntington’s disease, Parkinson’s disease, Mass spectrometry, Endogenous opioids

## Abstract

**Background:**

Proenkephalin (PENK) and prodynorphin (PDYN) are peptides mainly produced by the striatal medium spiny projection neurons (MSNs) under dopaminergic signaling. Therefore, they may represent candidate biomarkers in Huntington’s disease (HD) and Parkinson’s disease (PD), two neurodegenerative diseases characterized by striatal atrophy and/or dysfunction.

**Methods:**

Using an in-house established liquid chromatography−tandem mass spectrometry (LC–MS/MS) method in multiple reaction monitoring mode (MRM) we measured cerebrospinal fluid (CSF) levels of PENK- and PDYN- derived peptides in patients with HD (*n* = 47), PD (*n* = 61), Alzheimer’s disease (*n* = 11), amyotrophic lateral sclerosis (*n* = 14) and in 92 control subjects. Moreover, we investigated the possible associations between biomarkers and disease severity scales in HD and PD and the effect of dopaminergic therapy on biomarker levels in PD.

**Results:**

In HD, CSF PENK- and PDYN-derived peptide levels were significantly decreased compared to all other groups and were associated with disease severity scores. In PD, both biomarkers were within the normal range, but higher PDYN levels were found in dopamine-treated compared to untreated patients. In PD, both CSF PENK and PDYN did not correlate with clinical severity scales.

**Conclusions:**

CSF PENK- and PDYN-derived peptides appeared to be promising pathogenetic and disease severity markers in HD, reflecting the ongoing striatal neurodegeneration along with the loss of MSNs. In PD patients, CSF PDYN showed a limitative role as a possible pharmacodynamic marker during dopaminergic therapy, but further investigations are needed.

**Supplementary Information:**

The online version contains supplementary material available at 10.1007/s00415-022-11187-8.

## Introduction

Parkinson’s disease (PD) and Huntington’s disease (HD) are slowly progressive neurodegenerative diseases which share an impairment of striatal medium spiny projection neurons (MSNs) [1–3]. The latter play a role in the direct and indirect basal ganglia pathways by producing endogenous opioids under dopaminergic signaling [1–3]. In PD, the loss of nigrostriatal dopamine neurons induces a reduction in the number of dendritic spines on MSNs, whereas a selective neurodegeneration of MSNs occurs in HD [1, 2, 4, 5].

Endogenous opioids are a group of peptides that act on opioid receptors and derive from proteolytic cleavage of three main precursors: proenkephalin (PENK), prodynorphin (PDYN) and pro-opio-melanocortin (POMC) [6, 7]. Given their high expression in the striatum [6, 7], PENK and PDYN peptides might represent candidate biofluid markers reflecting striatal atrophy and/or dysfunction.

Using a newly established liquid chromatography−tandem mass spectrometry (LC–MS/MS) method in multiple reaction monitoring mode (MRM), we previously showed decreased cerebrospinal fluid (CSF) PDYN-derived peptide levels in HD and a tendency towards reduced levels in PD patients [8]. However, we did not investigate neither CSF PENK-derived peptides, which were also found to be reduced in HD [9, 10] nor the possible effect of dopaminergic therapy on CSF PENK and PDYN values in PD patients.

By addressing all these issues, we aimed here to evaluate CSF PENK- and PDYN-derived peptides as reliable candidate biomarkers in HD and PD. We also compared peptide levels in both diseases with those of controls and patients with amyotrophic lateral sclerosis (ALS) and Alzheimer’s disease (AD), which are neurodegenerative diseases characterized by lack (i.e. in the early stage) or low degree (i.e. in the middle-late stage) of striatal dysfunction/atrophy [11–13], to address the specificity of CSF PENK and PDYN-derived peptides as striatal atrophy/dysfunction markers. Moreover, we tested the possible associations between biomarker levels and disease severity scales in HD and PD and the influence of dopaminergic therapy on biomarkers levels in PD.

## Methods

### Patient selection

In the present study, we included 225 CSF samples collected from Ulm University Hospital (Germany) (*n* = 185) and from Section of Neurology, Perugia University Hospital (Italy) (*n* = 40): 47 patients with manifest HD, 61 with PD, 11 with AD, 14 with sporadic (s)ALS and 92 cognitively healthy non-neurodegenerative controls (Table 1).

**Table 1:** Demographic and clinical characteristics of the study cohort

	Controls	HD	PD	AD	sALS
*N*	92	47	61	11	14
Gender (males/females)	48/44	28/19	44/17	5/6	11/3
Age	52 (27–60)	52 (42–57)	68 (61–72)	66 (54–68)	64 (60–70)
Disease duration (years)	–	*n* = 36	*n* = 51	*n* = 11	*n* = 12
		4.2 (2.0–6.1)	3 (2–5)	2.5 (2–3)	1.8 (1.0–2.4)
UPDRS-part III	–	–	*n* = 60	–	–
			26 (20–31)		
Hoehn and Yahr scale	–	–	*n* = 60	–	–
			2 (2–2.5)		
MMSE	–	–	*n* = 61	–	–
			28 (26–29)		
MoCA	–	–	*n* = 39	–	–
			23 (20–26)		
CAG long	–	*n* = 47	–	–	–
		43 (42–46)			
CAG short	–	*n* = 47	–	–	–
		18 (17–20.5)			
Disease Burden Score	–	*n* = 45	–	–	–
		401 (357–481)			
UHDRS Total Motor Score	–	*n* = 43	–	–	–
		23(14.5–33.5)			
UHDRS Total Chorea Score	–	*n* = 43	–	–	–
		7 (5–9)			
UHDRS Cognitive Score	–	*n* = 39	–	–	–
		99 (72–183)			
UHDRS Total Functional Capacity	–	*n* = 47 (Stage 1: *n* = 31; 2: *n* = 7; 3: *n* = 6; 4: *n* = 3)	–	–	–
		12 (8.5–12)			

Values are given in median and interquartile ranges. *AD* Alzheimer’s disease, *HD* Huntington’s disease, *MMSE* Mini–Mental State Examination, *MoCA* Montreal Cognitive Assessment, *PD* Parkinson’s disease, *sALS* sporadic amyotrophic lateral sclerosis, *UHDRS* Unified Huntington's Disease Rating Scale, *UPDRS* unified Parkinson's disease rating scale

Clinical diagnoses of HD, PD, sALS and AD were made according to current diagnostic criteria [14–17]. Disease duration was assessed for all disease groups. For PD patients, we collected results from Unified Parkinson's Disease Rating Scale (UPDRS) [18], Hoehn and Yahr scale [18], Mini-Mental State Examination (MMSE) [19] and Montreal Cognitive Assessment (MoCA) [20] (Table 1). In HD patients, we assessed the following subscales of the Unified Huntington's Disease Rating Scale (UHDRS): Total Motor Score, Total Chorea Score (also called Chorea Sum Score), Cognitive Score and Total Functional Capacity (TFC) [21, 22]. Furthermore, in the same group, the Disease Burden Score was calculated according to the formula (CAGn-35.5) × age [23], whereas the TFC stage was obtained according to the TFC [24] (Table 1). The control group included 92 subjects lacking any clinical or neuroradiologic evidence of central nervous system disease.

The PD cohort encompasses cognitively healthy PD patients and PD patients with mild cognitive impairment (PD-MCI), according to Litvan et al. [25] To investigate the influence of dopaminergic therapy on biomarker levels, CSF samples from 21 and 19 PD-MCI patients from Perugia were analyzed separately after stratification into treated and untreated subgroups (Table 2). For these patients, levodopa daily dose (LDD) and levodopa equivalent daily dose (LEDD) were recorded [26].

**Table 2:** Levodopa administration in the PD and PD-MCI cohort from Perugia

	PD		PD-MCI	
	Treated	Untreated	Treated	Untreated
*N*	11	10	10	9
Gender (males/females)	6/5	8/2	9/1	6/3
Age	66 (59–69)	60 (51—63)	65 (62—69)	72 (64—72)
LDD (mg)	400 (275–510)	–	400 (300–500)	–
LEDD (mg)	400 (270–725)	–	500 (388—713)	–

Values are given in median and interquartile ranges. *LDD* Levodopa daily dose, *LEDD* levodopa equivalent daily dose, *PD* Parkinson’s disease, *PD-MCI* Parkinson’s disease with mild cognitive impairment

The study was approved by the local Ethics Committees of Ulm University (proposal number 20/10 and 259/09) and University of Perugia (CER Umbria 3944/21). All participants or their relatives gave written informed consent to participate in the study. The study has been performed in accordance with the ethical standards laid down in the 1964 Declaration of Helsinki and its later amendments.

### Measurement of CSF PENK and PDYN

CSF samples were obtained by lumbar puncture (LP) following a standard procedure, centrifuged and stored at − 80 °C. CSF PENK- and PDYN-derived peptides were analyzed in all cases using in-house established LC−MS/MS methods as described [8, 27]. Two hundred microliters of CSF sample were mixed with 12 µL internal standard solution containing heavy labelled peptides and 20 µL 1 M triethylammonium bicarbonate. Reduction and alkylation was conducted with 20 µL 1 M tris(2-carboxyethyl)phosphine and 2 µL 200 mM chloroacetamide for 10 min at 95 °C and 400 rpm. Samples were digested with 10 µL trypsin/Lys-C solution (0.1 µg/µL) for 18 h at 37 °C and 400 rpm. The reaction was stopped by adding trifluoroacetic acid (TFA) to a final concentration of 1%. Samples were fractionated using STAGE tips with increasing ammonium acetate concentrations in 20% acetonitrile (ACN)/0.5% formic acid (fraction 1–5: 125 mM, 160 mM, 220 mM, 300 mM and 450 mM). Eluates of fractions one, two and five were dried by vacuum centrifugation and dissolved in 27.5 µL 0.5% TFA/6% ACN for MS analysis.

We assessed CSF PENK and PDYN expression at the protein level based on the measurement of two PENK- and two PDYN-derived peptides, respectively [8, 27]. The four measured peptides have been named according to the respective letters of the first three and last three amino acids of the peptide sequence: PENK [DAE…LLK], PENK [FAE…YSK], PDYN [SVG…LAR] and PDYN [FLP…STR] [8, 27]. Further detailed LC–MS/MS methods are described elsewhere [8, 27] and in the Supplementary Table 1.

### Statistical analysis

The statistical analysis was performed using GraphPad Prism 7.0 (GraphPad Software, La Jolla, CA). CSF PDYN and PENK levels were compared between the disease groups by Kruskal–Wallis test and Dunn’s post hoc multiple comparison test. Two-way ANOVA and Bonferroni’s multiple comparison test was conducted to analyze the influence of levodopa treatment on biomarker levels in the PD/PD-MCI group. To assess significant associations between variables, the Spearman rank correlation coefficient was performed. For all analyses, *p* < 0.05 was considered statistically significant.

## Results

Groups were matched for gender (*p* = 0.0589) (Table 1). The control and HD group were significantly younger compared to the other disease groups (*p* < 0.0001).

**Fig. 1:**
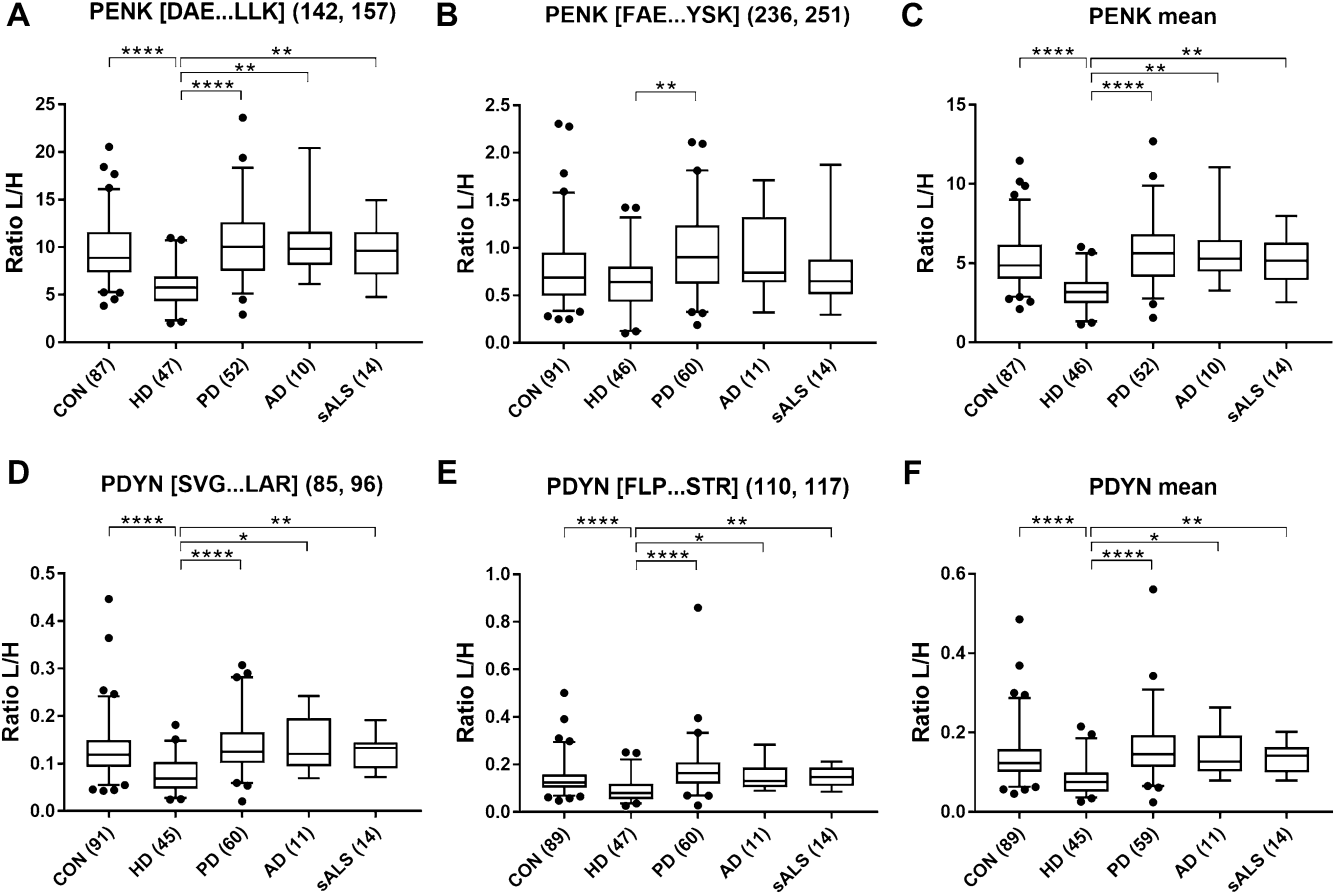
CSF levels of PENK and PDYN in HD, PD, AD, sALS and controls. Levels were determined by the measurement of two PENK-derived peptides (**A** [DAE…LLK], **B** [FAE…YSK], **C** mean values) and two PDYN-derived peptides (**D** [SVG…LAR], **E** [FLP…STR], **F** mean values) by liquid chromatography−tandem mass spectrometry (LC–MS/MS) method in multiple reaction monitoring mode (MRM). Median and interquartile range are shown for the ratio of light peptides to spiked heavy labelled peptides (L/H). Number of samples are shown in brackets. Kruskal–Wallis test and Dunn’s post hoc test (*p < 0.05, **p < 0.01, ****p < 0.0001). *CON* controls, *HD* Huntington’s disease, *PD* Parkinson’s disease, *AD* Alzheimer’s disease, *sALS* sporadic amyotrophic lateral sclerosis

CSF PENK [DAE…LLK] peptide and mean PENK values were significantly decreased in HD in comparison to the controls, PD, AD and sALS. PENK [FAE…YSK] peptide levels were significantly decreased in the HD group compared to PD patients. Both PDYN peptides, [SVG…LAR] and [FLP…STR], and PDYN mean values were decreased in HD patients compared to controls and all other disease groups (Fig. 1).

Weak-to-moderate correlations with age were determined with PENK [FAE…YSK] (*r* = 0.5191, *p* < 0.0001), PENK [DAE…LLK] (*r* = 0.2875, *p* = 0.0069), PENK mean (*r* = 0.3155, *p* = 0.0029), PDYN [SVG…LAR] (*r* = 0.2732, *p* = 0.0088) and PDYN mean (*r* = 0.2088, *p* = 0.0495) in the control group. Furthermore, CSF PENK [FAE…YSK] levels correlated with age in the PD (*r* = 0.3048, *p* = 0.0179) and sALS groups (*r* = 0.5692, *p* = 0.0366). No significant difference in biomarker levels between male and female participants were found in any group. Likewise, no association between biomarkers and disease duration was identified in any disease group (Supplementary Table 2).

In the PD group biomarkers levels were not associated with clinical scores. In the HD group, CSF PENK [DAE…LLK] correlated with the UHDRS Total Chorea Score (*r* = − 0.315, *p* = 0.0396). Moreover, both PDYN peptides, [SVG…LAR] (*r* = 0.471, *p* = 0.003) and [FLP…STR] (*r* = 0.3495, *p* = 0.029), and mean PDYN values (*r* = 0.400, *p* = 0.014) correlated with the UHDRS Cognitive Score in the HD group. CSF PDYN and PENK levels were not associated with TFC and TFC stage. No relationship was observed between biomarkers and the number of CAG repeats in the HD group.

**Fig. 2:**
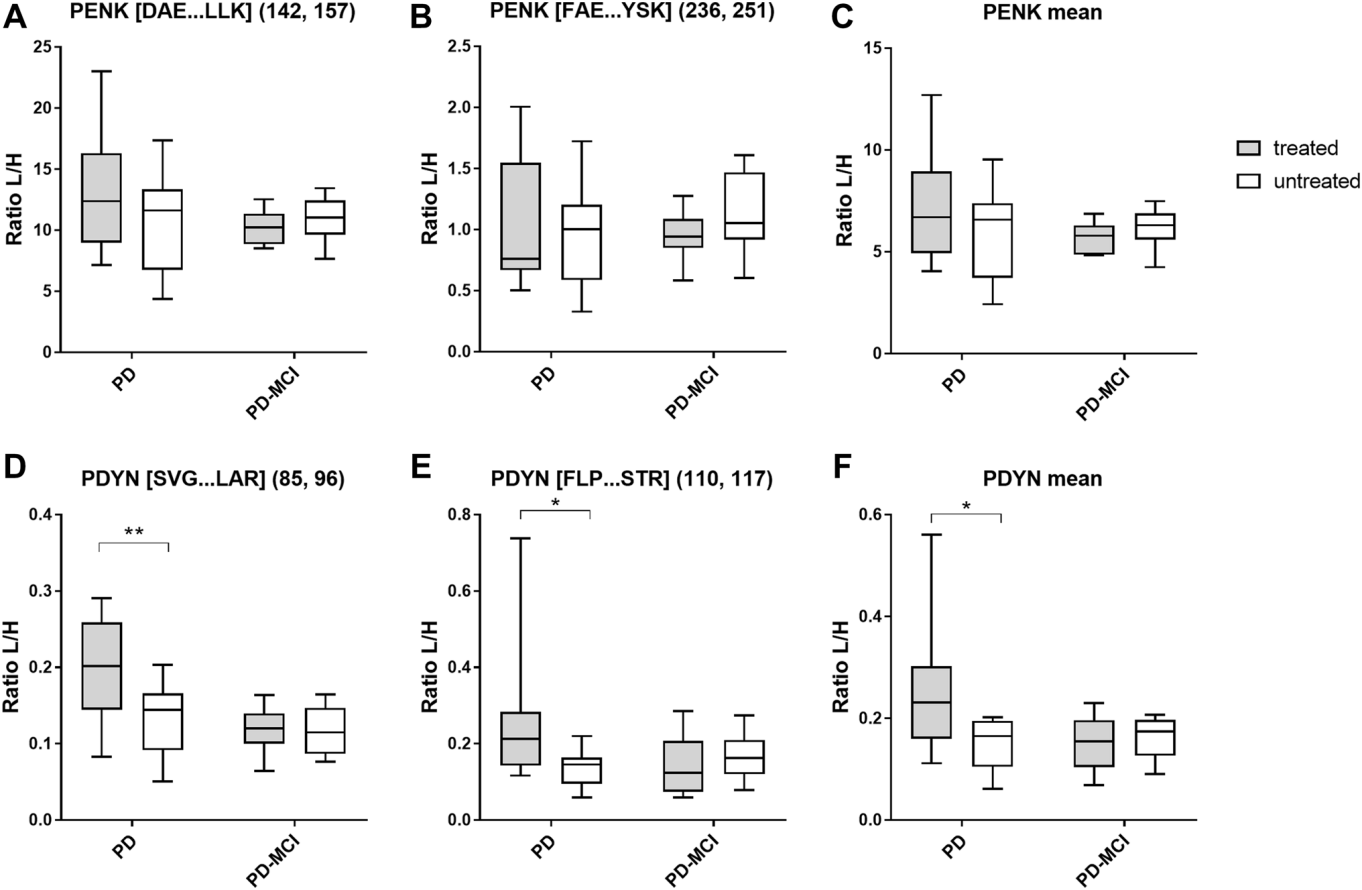
Influence of dopaminergic therapy on CSF PENK and PDYN levels. Levels were determined by the measurement of two PENK-derived peptides (**A** [DAE…LLK], **B** [FAE…YSK], **C** mean values) and two PDYN-derived peptides (**D** [SVG…LAR], **E** [FLP…STR], **F** mean values). Median and interquartile range are shown for the ratio of light peptides to spiked heavy labelled peptides (L/H). Two-way ANOVA and Bonferroni’s multiple comparisons test (*p < 0.05, **p < 0.01). *PD* Parkinson’s disease, *PD-MCI* Parkinson’s disease with mild cognitive impairment

The influence of levodopa or levodopa equivalent treatment on CSF PENK and PDYN levels was analyzed in a subgroup of PD and PD-MCI patients. CSF PENK levels were not significantly different in treated patients compared to untreated patients. In contrast, significantly higher PDYN-derived peptide and mean levels were observed in treated PD patients (Fig. 2). This effect was not seen in PD-MCI patients. No correlation with LDD or LEDD was observed for any peptide or group.

## Discussion

In the present study, we investigated CSF PENK- and PDYN- derived peptides as candidate biomarkers in PD and HD.

First, we added strength to our previous results [8] by showing reduced CSF levels of PDYN-derived peptides in an independent and larger cohort of HD patients in comparison to controls and other disease groups. Moreover, we found a similar profile for CSF PENK-derived peptides, in accordance with other studies [9, 10], thus supporting the notion that both CSF PENK and PDYN might reflect the striatal neurodegeneration along with the loss of MSNs occurring in HD [9, 10]. Consistently, a decrease in PDYN mRNA expression as well as in PDYN- and PENK-derived peptides has been reported in HD brains suggesting a combined effect of transcriptional dysregulation and loss of MSNs expressing *PENK* and *PDYN* genes [8, 9, 28–30]. Accordingly, CSF PENK and PDYN were within the normal range in ALS and AD, two neurodegenerative diseases with lack or low degree of striatal dysfunction/atrophy [11–13].

Interestingly, we found correlations between biomarker levels and both motor and cognitive parameters in HD patients, thus suggesting a potential role of both analytes as markers of disease severity in HD patients. Similarly, Niemela et al. [10] reported a decrease in CSF PENK levels along with increased disease severity, together with reduced biomarker concentrations in symptomatic compared to pre-symptomatic patients, and a trend toward lower levels in the latter group compared to controls. Given the urgent need for surrogate endpoints in ongoing clinical trials for HD, PDYN- and PENK-derived peptides may be used together with neurofilament light chain protein (NfL) as biomarkers of disease severity that could be potentially influenced by future disease-modifying therapies [31, 32]. In this regard, larger studies including longitudinal samples of HD symptomatic and pre-symptomatic subjects are needed to fully elucidate the dynamics of CSF PENK and PDYN levels during disease course and to evaluate their potential predictive role in the pre-symptomatic phase. Here, results in a small cohort documented an inverse correlation between CSF PENK levels and the 5-year risk of onset among pre-symptomatic HD cases [10]. We also acknowledge that the lack of association between biomarker levels and disease duration in HD patients might possibly depend on the relative homogeneity of our cohort (i.e. most HD patients in TFC stage 1).

On another issue, we did not find any biomarker changes in a large group of PD and PD-MCI subjects, compared to other diagnostic groups. Given that dopaminergic signaling modulates opioids synthesis by inducing PDYN and inhibiting PENK production, respectively [33], alterations in CSF biomarker levels were expected to be found in PD patients. However, animal model data showed that a subtotal striatal dopamine depletion should be a pre-requisite to produce a significant alteration in brain PDYN and PENK levels [34]. Thus, a possible explanation of our findings may rely on the inclusion of a relatively high proportion of PD patients in the early-middle disease stage (median disease duration 3 years).

Interestingly, the use of dopaminergic therapy influences considerably the production of endogenous opioids in PD animal models with an upregulation of PDYN and a downregulation of PENK, respectively [33, 35]. Accordingly, by comparing treated and untreated PD patients, we found higher CSF PDYN levels in the former compared to the latter group. However, this difference was not maintained in the PD-MCI group probably due to the advanced disease stage and the lower response to dopaminergic therapy. Similarly, CSF PENK levels were not altered in both PD and PD-MCI subjects after stratification according to the treatment state, suggesting possibly a less powerful effect of the therapy on CSF PENK levels.

The major strength of our study relies on the analysis of two new potential biomarkers in the largest cohort to date of HD patients. Regarding potential limitations, we would mention the cross-sectional nature of the study, which did not help in tracking the longitudinal evolution of biomarker values according to disease stage. Moreover, further clinical and therapeutical data (e.g. treatment duration and side effects, motor fluctuations) were not investigated in our cohort and deserve to be explored in future studies. The finding of positive associations between PENK peptide levels and age in controls, PD and sALS patients is challenging and deserves further explorations in bigger cohorts. Nevertheless, in all the above-mentioned groups both PENK-derived peptides were within the normal range and in PD, there was no correlation between biomarkers levels and disease severity scales, suggesting that age-related associations in PD and sALS may be driven by other pathophysiological phenomena compared to those of HD (i.e. striatal neurodegeneration). Furthermore, despite the very promising results, the main limit for the implementation of CSF PENK and PDYN analyses in the clinical diagnostic setting is the lower distribution of LC−MS/MS compared to classic ELISA techniques.

In conclusion, we provided further evidence on the performance of CSF PENK- and PDYN-derived peptides as promising candidate biomarkers reflecting ongoing striatal neurodegeneration and disease severity in HD. In PD patients, CSF PDYN showed a limitative role as a possible pharmacodynamic marker during dopaminergic therapy.

## Supplementary Information

Below is the link to the electronic supplementary material.

Supplementary file1 (DOCX 45 KB)
