# Supplementary material for: Cerebrospinal fluid levels of proenkephalin and prodynorphin are differentially altered in Huntington’s and Parkinson’s disease
Source: J Neurol. 2022 Jun 23;269(9):5136–43. doi: 10.1007/s00415-022-11187-8 (PMC9363351; doi:10.1007/s00415-022-11187-8)
Supplement: Supplementary file 1 — Supplementary file1 (DOCX 45 KB) [file 415_2022_11187_MOESM1_ESM.docx]

**Supplementary materials**

**Supplementary Table 1: MRM methods**

| Fraction 1 | | | | | | | | | |
| --- | --- | --- | --- | --- | --- | --- | --- | --- | --- |
| Precursor mass | Product ion mass | RT (min) | Peptide | DP | EP | CE | CXP | ST1 |  |
| 861.39 | 960.54 | 7.6 | sp\|P01210\|PENK_HUMAN.DAEEDDSLANSSDLLK.+2y9.light | 90 | 11 | 42.9 | 49 | -22 |  |
| 816.39 | 776.41 | 7.6 | sp\|P01210\|PENK_HUMAN.DAEEDDSLANSSDLLK.+2y7.light | 90 | 11 | 42.9 | 41 | -22 |  |
| 816.39 | 316.11 | 7.6 | sp\|P01210\|PENK_HUMAN.DAEEDDSLANSSDLLK.+2b3.light | 90 | 11 | 42.9 | 20 | -22 |  |
| 865.40 | 968.55 | 7.6 | sp\|P01210\|PENK_HUMAN.DAEEDDSLANSSDLLK.+2y9.heavy | 90 | 11 | 42.9 | 49 | -22 |  |
| 865.40 | 784.43 | 7.6 | sp\|P01210\|PENK_HUMAN.DAEEDDSLANSSDLLK.+2y7.heavy | 90 | 11 | 42.9 | 41 | -22 |  |
| 865.40 | 316.11 | 7.6 | sp\|P01210\|PENK_HUMAN.DAEEDDSLANSSDLLK.+2b3.heavy | 90 | 11 | 42.9 | 20 | -22 |  |
| 879.89 | 1227.50 | 6.1 | sp\|P01210\|PENK_HUMAN.FAEALPSDEEGESYSK.+2y11.light | 90 | 11 | 43.7 | 55 | -22 |  |
| 879.89 | 348.16 | 6.1 | sp\|P01210\|PENK_HUMAN.FAEALPSDEEGESYSK.+2b3.light | 90 | 11 | 43.7 | 21 | -22 |  |
| 879.89 | 419.19 | 6.1 | sp\|P01210\|PENK_HUMAN.FAEALPSDEEGESYSK.+2b4.light | 90 | 11 | 43.7 | 24 | -22 |  |
| 883.90 | 1235.52 | 6.1 | sp\|P01210\|PENK_HUMAN.FAEALPSDEEGESYSK.+2y11.heavy | 90 | 11 | 43.7 | 55 | -22 |  |
| 883.90 | 348.16 | 6.1 | sp\|P01210\|PENK_HUMAN.FAEALPSDEEGESYSK.+2b3.heavy | 90 | 11 | 43.7 | 21 | -22 |  |
| 883.90 | 419.19 | 6.1 | sp\|P01210\|PENK_HUMAN.FAEALPSDEEGESYSK.+2b4.heavy | 90 | 11 | 43.7 | 24 | -22 |  |
| Fraction 2 | | | | | | | | | |
| Precursor mass | Product ion mass | RT (min) | Peptide | DP | EP | CE | CXP | ST1 |  |
| 618.81 | 1050.51 | 5.5 | sp\|P01213_1\|PDYN_HUMAN.SVGEGPYSELAK.+2y10.light | 90 | 10 | 32.2 | 53 | -22 |  |
| 618.81 | 864.45 | 5.5 | sp\|P01213_1\|PDYN_HUMAN.SVGEGPYSELAK.+2y8.light | 90 | 10 | 32.2 | 44 | -22 |  |
| 618.81 | 807.42 | 5.5 | sp\|P01213_1\|PDYN_HUMAN.SVGEGPYSELAK.+2y7.light | 90 | 10 | 32.2 | 42 | -22 |  |
| 618.81 | 432.73 | 5.5 | sp\|P01213_1\|PDYN_HUMAN.SVGEGPYSELAK.+2y8+2.light | 90 | 10 | 32.2 | 25 | -22 |  |
| 622.82 | 1058.52 | 5.5 | sp\|P01213_1\|PDYN_HUMAN.SVGEGPYSELAK.+2y10.heavy | 90 | 10 | 32.2 | 53 | -22 |  |
| 622.82 | 872.46 | 5.5 | sp\|P01213_1\|PDYN_HUMAN.SVGEGPYSELAK.+2y8.heavy | 90 | 10 | 32.2 | 45 | -22 |  |
| 622.82 | 815.44 | 5.5 | sp\|P01213_1\|PDYN_HUMAN.SVGEGPYSELAK.+2y7.heavy | 90 | 10 | 32.2 | 42 | -22 |  |
| 622.82 | 436.73 | 5.5 | sp\|P01213_1\|PDYN_HUMAN.SVGEGPYSELAK.+2y8+2.heavy | 90 | 10 | 32.2 | 25 | -22 |  |
| Fraction 5 | | | | | | | | | |
| Precursor mass | Product ion mass | RT (min) | Peptide | DP | EP | CE | CXP | ST1 |  |
| 446.76 | 745.45 | 6.2 | sp\|P01213_1\|PDYN_HUMAN.FLPSISTK.+2y7.light | 75 | 10 | 24.7 | 29 | -14 |  |
| 446.76 | 632.36 | 6.2 | sp\|P01213_1\|PDYN_HUMAN.FLPSISTK.+2y6.light | 75 | 10 | 24.7 | 34 | -14 |  |
| 446.76 | 535.31 | 6.2 | sp\|P01213_1\|PDYN_HUMAN.FLPSISTK.+2y5.light | 75 | 10 | 24.7 | 30 | -14 |  |
| 446.76 | 316.68 | 6.2 | sp\|P01213_1\|PDYN_HUMAN.FLPSISTK.+2y6+2.light | 75 | 10 | 24.7 | 20 | -14 |  |
| 450.77 | 753.46 | 6.2 | sp\|P01213_1\|PDYN_HUMAN.FLPSISTK.+2y7.heavy | 75 | 10 | 24.7 | 40 | -14 |  |
| 450.77 | 640.38 | 6.2 | sp\|P01213_1\|PDYN_HUMAN.FLPSISTK.+2y6.heavy | 75 | 10 | 24.7 | 34 | -14 |  |
| 450.77 | 543.32 | 6.2 | sp\|P01213_1\|PDYN_HUMAN.FLPSISTK.+2y5.heavy | 75 | 10 | 24.7 | 30 | -14 |  |
| 450.77 | 320.69 | 6.2 | sp\|P01213_1\|PDYN_HUMAN.FLPSISTK.+2y6+2.heavy | 75 | 10 | 24.7 | 20 | -14 |  |

*RT* retention time, *DP* declustering potential, *EP* entrance potential, *CE* collision energy, *CXP* cell exit potential, *ST1* prefilter

**Supplementary Table 2: Correlations between disease duration and biomarker levels in the disease groups.**

|  | **HD** | **PD** | **AD** | **sALS** |
| --- | --- | --- | --- | --- |
| **N** | 36 | 51 | 11 | 12 |
| **PENK [DAE…LLK]**  **L/H ratio** | r = 0.186  p = 0.277 | r = -0.001  p = 0.994 | r = -0.219  p = 0.518 | r = -0.091  p = 0.778 |
| **PENK [FAE…YSK]**  **L/H ratio** | r = 0.309  p = 0.067 | r = 0.060  p = 0.681 | r = -0.558  p = 0.074 | r = 0.004  p = 0.991 |
| **Mean PENK**  **L/H ratio** | r = 0.229  p = 0.180 | r = -0.016  p = 0.922 | r = -0.489  p = 0.151 | r = -0.165  p = 0.609 |
| **PDYN [SVG…LAR]**  **L/H ratio** | r = -0.053  p = 0.760 | r = 0.083  p = 0.569 | r = -0.016  p = 0.962 | r = -0.007  p = 0.983 |
| **PDYN [FLP…STR]**  **L/H ratio** | r = -0.081  p = 0.640 | r = 0.007  p = 0.959 | r = -0.093  p = 0.786 | r = 0.056  p = 0.862 |
| **Mean PDYN**  **L/H ratio** | r = -0.039  p = 0.828 | r = 0.042  p = 0.778 | r = -0.087  p = 0.095 | r = 0.049  p = 0.879 |

*AD* Alzheimer’s disease, *HD* Huntington’s disease, *PD* Parkinson’s disease, *sALS* sporadic amyotrophic lateral sclerosis
